# Supplementary material for: From Metabolomics to Function: Ranking Plant Stem Cell Metabolomes for Use in Health and Cosmetics
Source: Biomolecules. 2026 May 7;16(5):695. doi: 10.3390/biom16050695 (PMC13204304; doi:10.3390/biom16050695)
Supplement: Supplementary file 1 [file biomolecules-16-00695-s001.zip › Supplementary Table S1.pdf]

Table S1. Functions and keywords. Metabolite names and keywords were used to search PubMed database for relevant articles.

**Function**	**Keywords**
anti-aging	anti-aging, anti-ag*, antiag*, aging, age, longevity
anti-glycation	glycation, AGE-protein, AGE-formation
anti-inflammatory	anti-inflamma*, antiinflamma*, inflammat*
antimicrobial	antimicrobial, antimicrob*, antibiotic, antifung*, antiparasitic, anti-bacteria*, antibacteria*
antioxidant	antioxid*, anti-oxid*, oxidation, reactive oxygen species
anti-senescence	senescence, senolytic, telomere, antisenescence, anti-senescence
anti-wrinkle	anti-wrinkl*, antiwrinkl*, wrinkl*
collagen	collagen, procollagen, matrix metalloproteinases
elastin	elastin, antitrypsin, elastase*
hyaluronic acid	hyaluronic acid
skin care	skin
skin lightening	skin AND pigment*, tyrosinase, pigment*, melanin, melanogenesis
