# Supplementary material for: From Metabolomics to Function: Ranking Plant Stem Cell Metabolomes for Use in Health and Cosmetics
Source: Biomolecules. 2026 May 7;16(5):695. doi: 10.3390/biom16050695 (PMC13204304; doi:10.3390/biom16050695)
Supplement: Supplementary file 1 [file biomolecules-16-00695-s001.zip › Supplementary Table S2.pdf]

Table S2. Function Prompts. Prompts used to annotate functions to metabolites using GPT model 4.1-mini.

**Function**	**GPT Prompt**
Anti-aging	Follow the decision with **one short sentence (≤30 words)** explaining whether the metabolite has anti-aging properties. State the metabolite name, the biomarker measured (e.g., collagen levels, MMP-1 inhibition), and the observed effect on aging markers.
Anti-glycation	Follow the decision with **one short sentence (≤30 words)** naming the metabolite, assay/model (e.g., BSA–glucose/fructose, MGO/GO trapping, fructosamine, fluorescent AGEs, CML/pentosidine, collagen/elastin crosslinks, or others), the direction of change of glycation, and—if explicit—the mechanism (Schiff-base competition, carbonyl trapping, lysine masking/shielding, anti-aggregation, ion-chelation, insulin-axis). Clarify prevention (anti-glycation) vs reversal (transglycation).
Anti-inflammatory	Follow the decision with **one short sentence (≤30 words)** explaining whether the metabolite has anti-inflammatory properties. Mention the metabolite name, the inflammatory marker (e.g., TNF-α, IL-6, COX-2), and the in vitro or in vivo model used and how it decreased inflammation.
Antimicrobial	Follow the decision with **one short sentence (≤30 words)** explaining whether the metabolite has antimicrobial properties. State the metabolite name, the organism or strain tested, the assay type (e.g., MIC, zone of inhibition), and whether it inhibited or killed microorganisms (bacteria, viruses, fungi, protozoa).
Antioxidant	Follow the decision with **one short sentence (≤30 words)** explaining whether the metabolite increases antioxidant activity. Mention the metabolite name, the specific assay (e.g., DPPH, ABTS, FRAP, ORAC, TBARS, DCFH-DA), the model system, and whether it scavenged free radicals or reduced oxidative damage.
Anti-senescence	Follow the decision with **one short sentence (≤30 words)** explaining whether the metabolite inhibits or suppresses cellular senescence. State the metabolite name, the senescence marker (e.g., β-galactosidase, p16^INK4a), the model system, and the observed effect on senescent cell burden.
Anti-wrinkle	Follow the decision with **one short sentence (≤30 words)** explaining whether the metabolite has anti-wrinkle properties. Mention the metabolite name, the skin assay or model (e.g., wrinkle depth measurement, elastase inhibition), and the observed outcome.
Collagen	Follow the decision with **one short sentence (≤30 words)** explaining whether the metabolite increases collagen synthesis or reduces its degradation. Include the metabolite name, the assay or marker (e.g., hydroxyproline, fibroblast collagen production), and the beneficial effect on collagen.
Elastin	Follow the decision with **one short sentence (≤30 words)** explaining whether the metabolite improves elastin synthesis or inhibits its degradation. Include the metabolite name, the assay or model (e.g., elastase inhibition, elastin immunostaining), and the observed effect on elastin.
Hyaluronic acid	Follow the decision with **one short sentence (≤30 words)** explaining whether the metabolite increases hyaluronic acid synthesis or reduces its degradation. Mention the metabolite name, the assay or cell model, and the directional change in hyaluronic acid levels.
Skin care	Follow the decision with **one short sentence (≤30 words)** explaining whether the metabolite improves skin properties. Mention the metabolite name, the clinical or in vitro assay, and the observed benefit to skin condition.
Skin lightening	Follow the decision with **one short sentence (≤30 words)** explaining whether the metabolite has skin lightening properties. Include the metabolite name, the melanogenesis or tyrosinase inhibition assay, and the observed effect on pigmentation.
